# Supplementary material for: Low-degree mantle melting controls the deep seismicity and explosive volcanism of the Gakkel Ridge
Source: Nat Commun. 2022 Jun 3;13:3122. doi: 10.1038/s41467-022-30797-4 (PMC9166806; doi:10.1038/s41467-022-30797-4)
Supplement: Supplementary file 1 — Supplementary Information [file 41467_2022_30797_MOESM1_ESM.pdf]

**Supporting information for the paper "Low-degree mantle melting controls deep seismicity and explosive volcanism of the Gakkel Ridge" by Koulakov I., Schlindwein V., Liu M., Gerya T., Jakovlev A. and Ivanov A. submitted to *Nature Communications*.**

## 1. General information about Gakkel Ridge and the volcanic area at 85°E

The opening of the Arctic Ocean presently occurs along the ~1800 km long Gakkel Ridge separating the Eurasian and North American Plates causing the forming of a large deep water Eurasian Basin (Fig. 1a). Gakkel Ridge is characterized by an ultraslow divergence rate ranging from 13 mm/year in the western part to 6.3 mm/year near the eastern end in the Laptev Sea (1,2). Such slow values of the spreading velocities are compatible with only a few locations in the world, such as Mohn and Knipovich Ridges (15-17 mm/year) (60) and the Southwest Indian Ridge (~14 mm/year) (3,61). In most spreading centers in other oceans, the divergence rates exceed 20-30 mm/year and sometimes reaches 150 mm/year (62,3). Despite such a slow spreading of the Gakkel Ridge, the oceanic lithosphere in the Eurasian Basin clearly demonstrates fairly well developed linear magnetic anomalies similar to ones observed around fast spreading centers.

Gakkel Ridge is exceptionally deep compared to other spreading centers. The sea bottom along the axial valley reaches depths of more than 5000 m (3), whereas in other spreading centers, the depth rarely reaches 4000 m, and usually at around 3000-3500 m (4). Another feature of the Gakkel Ridge is that its axis line is smooth and almost not dissected by transform faults (3). Such a behavior is only observed on a few other ultra-slow ridges (such as Mohn and Knipovich Ridges), but it appears to be exceptional compared to other spreading centres.

Gakkel Ridge manifests high seismic activity with a large number of strong earthquakes with the magnitudes of more than 3, which were recorded for decades by global seismic stations (5). Seismic networks deployed recently on ice floes have revealed that some of the earthquakes beneath Gakkel Ridge occurred at mantle depths of up to 25 km (7) similarly as in other slow-spreading centers, such as in Southwest Indian Ridge (8) and Knipovich Ridge (9). However, the depth of the brittle-ductile boundary varies strongly along the axis with the deepest earthquakes occurring mostly in magma starved areas away from recently active volcanic centres (62,63). Therefore, the deep seismicity appears to be a

common feature for slow spreading caused by anomalously low temperature beneath the rift axis (65,10).

Finally, in some locations, the Gakkel Ridge is characterized by high volcanic and geothermal activity (11,12). This seems to contradict a classical model of functioning the mid-oceanic ridge presuming low magma supply in cases of slow spreading centers (63, 64). On the other hand, these areas of exceptionally strong volcanism along the Gakkel Ridge are alternated with large segments without significant traces of magmatic activity, thus on average, the total magma supply is rather low. The reason of such alternation is not yet understood. Traces of explosive volcanic activity indicate that the magmas are highly contaminated with volatiles, which is not typical for mid-oceanic basalts.

Here we focus on a volcanic area located at the 85°E segment of the Gakkel Ridge, which is morphologically composed of a 12-15 km wide rift valley and several linear volcanic ridges (Fig. 1c). One of the lineaments includes Jessica’s and Duque’s Hills, and another is formed of three distinct volcanoes Oden, Loke and Thor together with a series of smaller volcanic cones (16, 18).

In 1999, an exceptionally strong seismicity swarm struck in this area including several hundreds of earthquakes with the magnitudes exceeding M3.2 and reaching M5.8 recorded for seven months. Focal mechanisms of these events indicated that this swarm might be associated with dyke intrusions due to magmatic activity (13, 14, 15).

Immediately after the crisis, a sidescan sonar surveying of during the SCICEX expedition on board a nuclear-powered submarine revealed large areas of high acoustic reflectivity interpreted as fresh non-consolidated lava flows and pyroclasts (17). In 2001, the AMORE expedition, which was based on the German and USA research icebreakers, detected a massive hydrothermal megaplume in the water column at 85°E volcanic centre suggesting ongoing volcanic activity (12). During this campaign in 2001, the first attempts to deploy seismic stations on ice floes were conducted. The array of four seismic stations was mounted on a single ice floe and operated for 11 days. It has allowed recording local seismic events possibly representing seismoacoustic explosion sounds having volcanic nature, as well as several regional earthquakes (65, 19). It is important that the results of this campaign have proven for the first time that the seismic network on ice floes is capable to record clear

arrivals of both P and converted to SP waves, and it can be used for determination of hypocentral parameters for the local seismicity.

More evidence of recent effusive and explosive eruptions in this area were obtained during the next field campaign in this area called AGAVE conducted in 2007 with the icebreaker Oden. The detailed bathymetry derived during this expedition (Fig. 2c) has demonstrated a morphology of the active volcanic cones (16). This expedition was equipped with submarine video cameras and dredging instruments that provided the details of the seafloor morphology and gave the evidence of effusive and explosive eruptions and fresh lava flows within ~12-15 km-wide axial valley. They found that the source vents of the recent eruption were most likely located on the Oden and Loke volcanoes and consisted of relatively small lava flows and pyroclasts, which were ejected during a series of powerful vulcanian-type eruptions (18). These manifestations of recent volcanic activity coincided with the location of thermal anomaly in the water layer identified in 2001 (12) and with observations of microbial mats on the seafloor.

The identified evidence for magma fragmentation due to explosive eruptions presume a large amount of volatiles in magmas and their intensive degassing during eruptions. In particular, Sohn et al. (16) provided video records of unconsolidated pyroclastic deposits composed of bubble wall fragments on the seafloor at the depth of ~4000 m. Such structures are very atypical for mid-oceanic ridges, and have never been previously observed on a sea floor below 3000 m depth. Sohn et al. (16) proposed that magma fragmentation at such depths is possible only in a case of a large amount of volatiles in deep-seated magma storages and estimated that at least 13 wt% of $CO_2$ is necessary to run the degassing process at such depths. This is much higher than estimated values of dissolved volatiles in most of the mid-ocean ridge basalts (~1.4 wt% on average).

During the AGAVE campaign, a seismic network was deployed on the ice floes in the study area, and it recorded signals from hundreds of local events. The arrival times of the *P*-waves were used to build a preliminary tomography model (20). However, as seen in Fig. S1 of supporting materials, the existing seismic records demonstrate clear arrivals of secondary phases, which are interpreted as converted *SP*-waves. The possibility of recording such waves by seismic stations on ice floes was supported by numerical modeling of full seismic wavefields (21). In the present study, we revisit this dataset and pick the *SP*-wave data. As a result, we present a new tomographic model including the *P* and *S* wave velocity

distributions. The additional information on the *S*-wave velocity, and especially on the Vp/*Vs* ratio, is crucial for revealing the sources of magmatic activity.

## 2. Seismic observations and tomography inversion

Seismic data for this study were collected in a field campaign called AGAVE, which was conducted in 2007 by the German icebreaker Oden in the area of the 85°E zone. The details of this seismic experiment and data processing workflow were described in (20). The seismic network was installed in the area of the recent volcanic activity and operated for 16 days from July 15 to July 31 2007. The network consisted of three arrays deployed on separate ice floes. Each array was composed of four seismometers installed together on a single ice floe at a distance of ~1 km from each other. The arrays floated over the rift valley, and when they left the area of interest, the stations were taken off and redeployed on another ice floe. In this way, each array was redeployed two times. In total, this network included 12 short period Mark 4-4C3D seismometers that provided three-component seismograms with the sampling rate of 100 Hz. Once per hour, every station recorded the current location coordinates. The locations of the stations in the moment of an event were determined by linear interpolation. The movement tracks of the seismic stations are shown in Fig. 1 of the main article.

To detect the earthquake-generated signal, the vertical components were used; however, the other components were important to distinguish them from the ice-quakes that were mostly expressed on the horizontal components. To pick the arrival times from the local seismicity, we used the bandpass filtering in the frequency range between 5 and 15 Hz. During manual picking, we have carefully inspected all seismograms to exclude phase misidentification. Multiples within the water column could easily be distinguished from SP converted phases. Since the seafloor is about 4 km deep in the survey area, water multiples arrive about 6 s after the P phase. Epicentral distances in this local survey are less than 50-60 km such that a confusion is unlikely as SP phases arrive before the multiple. In addition, the water multiple is only seen clearly at short epicentral distances. The SP converted phase is usually clearly visible as in this example when the SP phase arrives later than the P coda. For close-by events, the SP phase is detected within the P coda and not necessarily larger in amplitude than the P phase, as in this example. Since in these cases, the SP phase is difficult to identify, only phases were picked that appeared on at least 2 of the array stations. After initial picking, all events were located and each location output file was inspected

individually and each phase residual checked. Spurious arrivals with large residuals were removed or repicked. In this way, each phase of the entire data set was checked. Correctly fitting P phase arrivals was given a high priority. In addition, since we were using mini-arrays, the event azimuth was also checked against the propagation direction across the mini-array. In this way, we have confidence in our phase picks and could locate events also when only one array out of three provided S phases. An example of picking an event is presented in Fig. S1, which demonstrates that the converted S-waves are clearly detectable in most seismic traces.

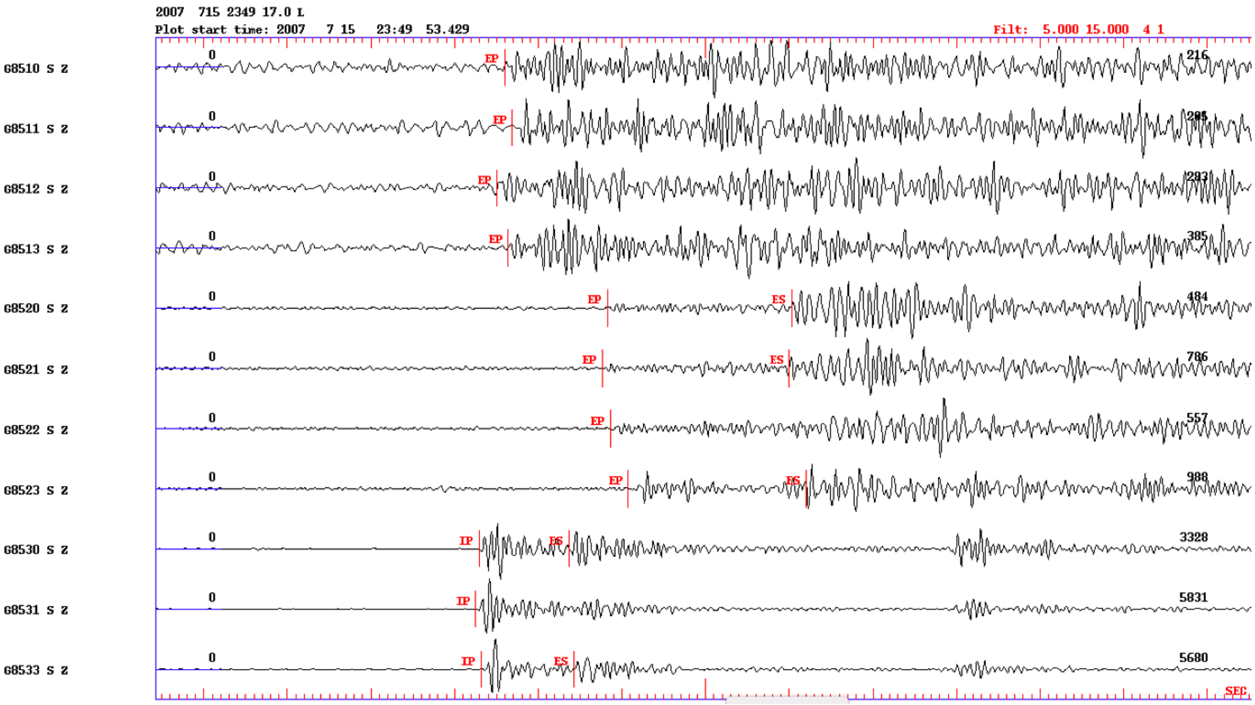


**Figure S1. Example of vertical component records** for 11 seismic stations with picking the arrival times of the P and converted SP waves from a local event.

In total, we identified 259 local earthquakes and picked 1722 *P* phases and 631 *PS* phases. However, for tomography, we selected the events with 8 or more picks, which resulted in reduction of the dataset to 117 sources with 1028 *P* and 498 *S* wave arrival times.

For tomography, we used a version of the LOTOS code (22), which was specially adapted for a case of a floating network. The details of the algorithm are described in the Method section.

Some modifications had to be done for the input format, which included the variable coordinates of the stations for each event. Both the *P* and *S* wave velocity models included

the water layer with a constant velocity value equal to 1.44 km/s. The ray tracing was conducted by a 3D bending algorithm for the entire path between source and receiver including the water layer. For calculation of travel times, we used lower resolution regional bathymetry model presented in Figure 1c, which appears to be adequate regarding some natural averaging occurring due to the finite frequency content of the arriving waves. For example, for periods of 0.2-0.5 s and velocity 5-7 km/s, the wavelength would be in the range of 1-2 km. Therefore, the conversion of seismic waves on the sea bottom will occur not in a point, but in an area of ~1 km size. For this reason, the converted travel times would not be strongly affected by high-resolution patterns

Note that in the ocean water, there are some velocity changes of a few percent in a range of 1490-1520 m/s (66) that may affect travel times of seismic rays. The major changes occur in the vertical direction due to depth stratification of salinity and temperature. If we knew the actual 1D velocity profile in the water and could use it to calculate more accurately the rays, the travel times would be biased in one direction to approximately same value. Regarding the uncertainty of the origin time determinations of seismic events, this nearly constant bias would not affect the results. The role of lateral heterogeneities cannot be estimated, because their distribution is impossible to identify.

Note that the P-wave data from approximately same dataset were previously used to build a tomography model by Korger and Schlindwein (20). However, in their work, the source locations were determined at an initial step and then remained fixed in the following inversions. The LOTOS code provides much more sophisticated workflow, in which both P and S waves are incorporated and the locations of sources are performed simultaneously with velocity model determinations at all iteration steps. This enables much higher accuracy for the source and velocity solutions.

In total, we performed five iterations including relocation and inversion steps. The values of the mean residuals in the L1 norm and their reduction during the iterative inversions are presented in Table S1. It can be seen that the minimum residuals are obtained after the iteration 4; then in the 5th iteration, the mean residuals slightly increase. Therefore, for the main model, we used the inversion results after the 4th iteration. Note that the strongest reduction is obtained for the S-wave residuals, which can be explained by higher sensitivity of S-waves travel times to the distributions of melts and fluids, which are presumed to be the main factor of heterogeneities in volcanic areas. The distributions of the residuals after the 1st

and 4th iterations are also presented in histograms in Figure S2, which demonstrate their clear narrowing during the inversions.

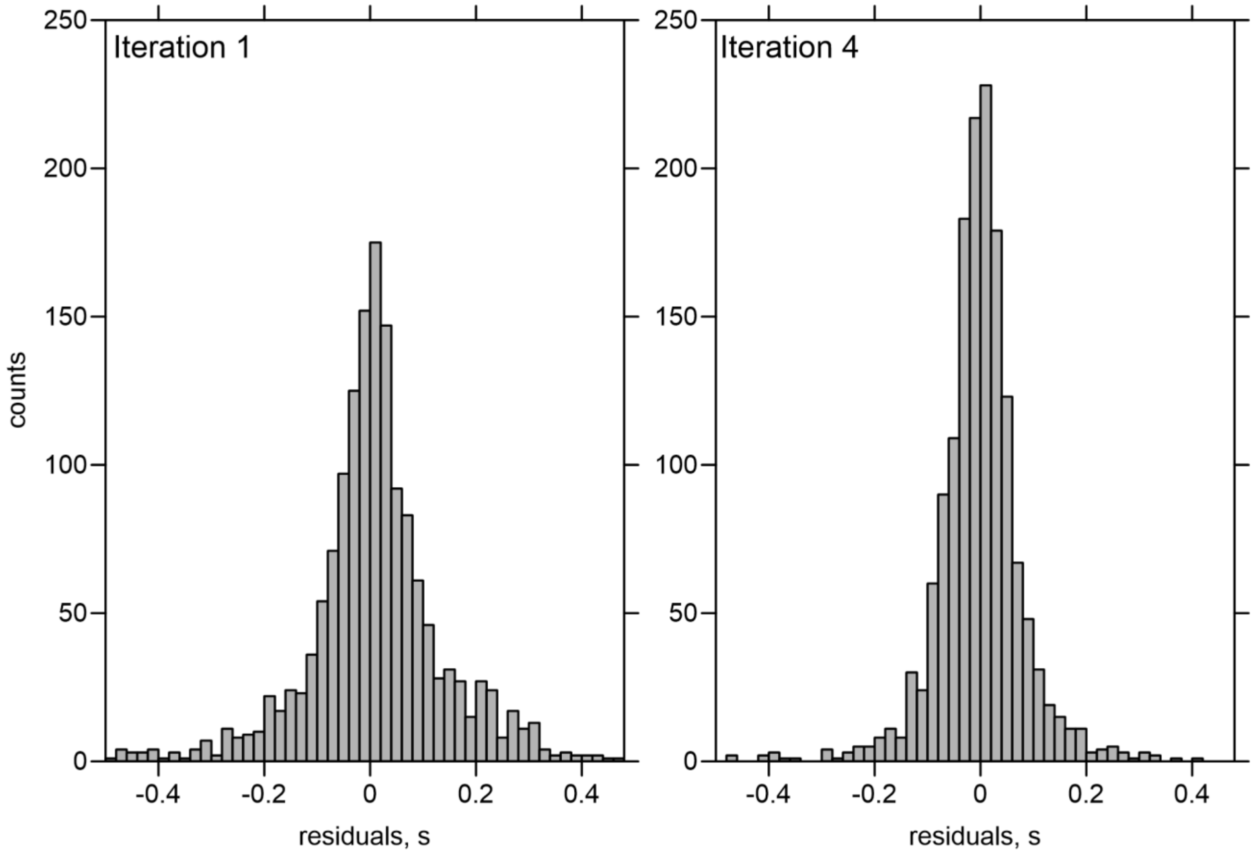


Figure S2. Histograms of the time residuals corresponding to the starting velocity model (iteration 1) and the final 3D velocity model (iteration 4).

**Table S1.** Values of the *P* and *S* wave residuals and their reduction during the iterative tomographic inversion of the experimental data

| iteration | dt *P*, s | reduction, dt *P*, % | dt *S*, s | reduction, dt *S*, % |
|---|---|---|---|---|
| 1 | 0.076 | 0.00 | 0.124 | 0.00 |
| 2 | 0.066 | 12.30 | 0.066 | 46.40 |
| 3 | 0.056 | 25.20 | 0.064 | 48.20 |

| | | | | |
|---|---|---|---|---|
| 4 | 0.054 | 28.46 | 0.060 | 51.03 |
| 5 | 0.056 | 26.15 | 0.063 | 48.92 |

As a result of the iterative tomography inversions, we have obtained the 3D distributions of the P- and S-wave velocities as well as coordinates of events relocated in the derived 3D models, which are presented in two horizontal and two vertical sections in Figs. S3 and S4. The distribution of the P-wave velocity anomalies in the new tomography model demonstrates some similar features compared to the previous model (20). At the same time, some changes in the velocity model are caused by considerable improvement in source location accuracy owing to adding the S-wave data and simultaneous inversions for the velocity and source parameters.

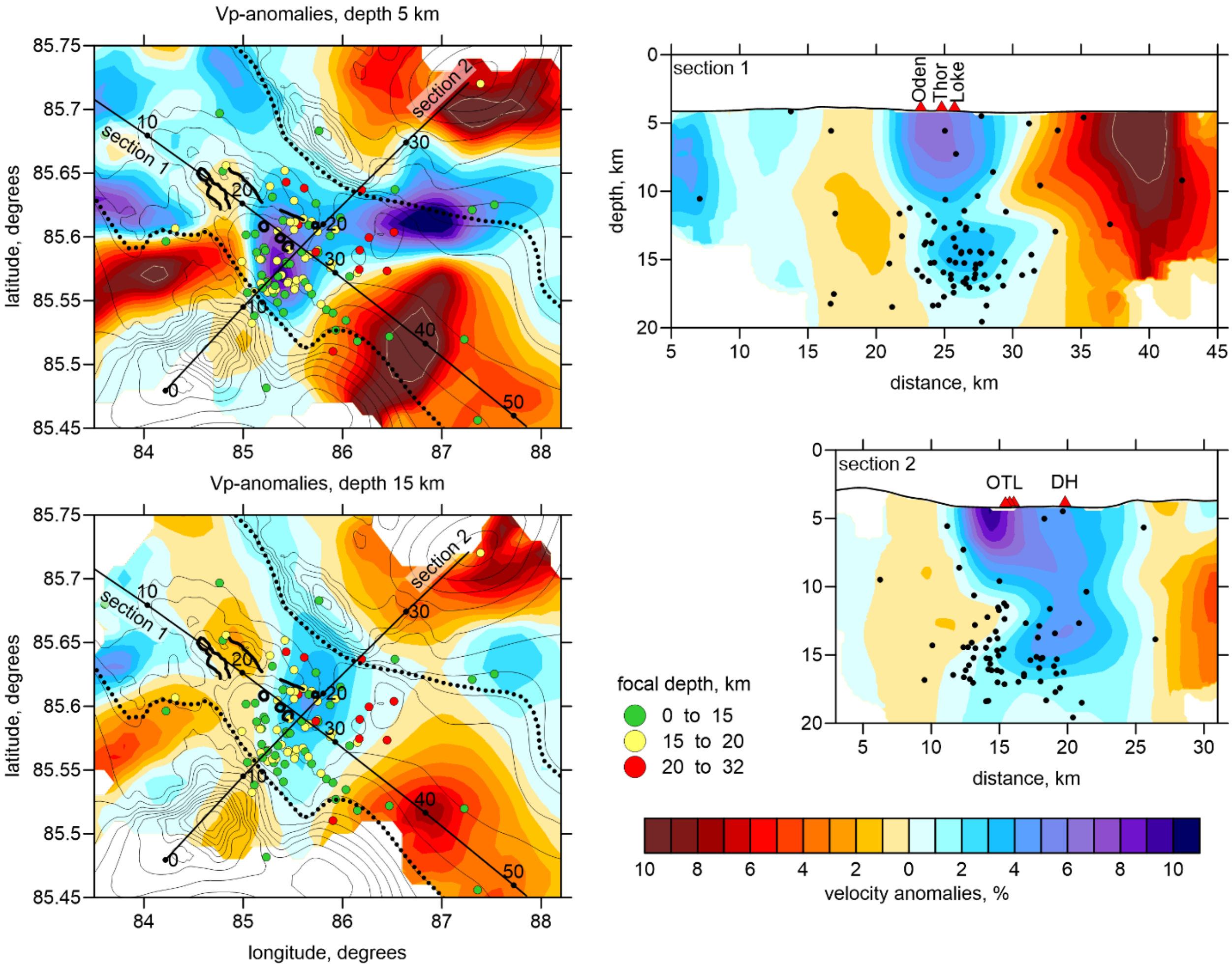


**Figure S3. The resulting distributions of the Vp anomalies** in two horizontal and two vertical sections. Colored dots in the horizontal sections indicate the locations of earthquakes ranged by

depth. Dotted lines indicate the borders of the rift valley. The major volcanic structures are highlighted with solid black lines. The bathymetry is presented by thin contour lines at every 100 m. In the vertical sections, black dots indicate the locations of the events at the distances less than 7 km. Red triangles depict the volcanic structures (OTL – Oden, Thor, Loke; DH – Duque's Hills).

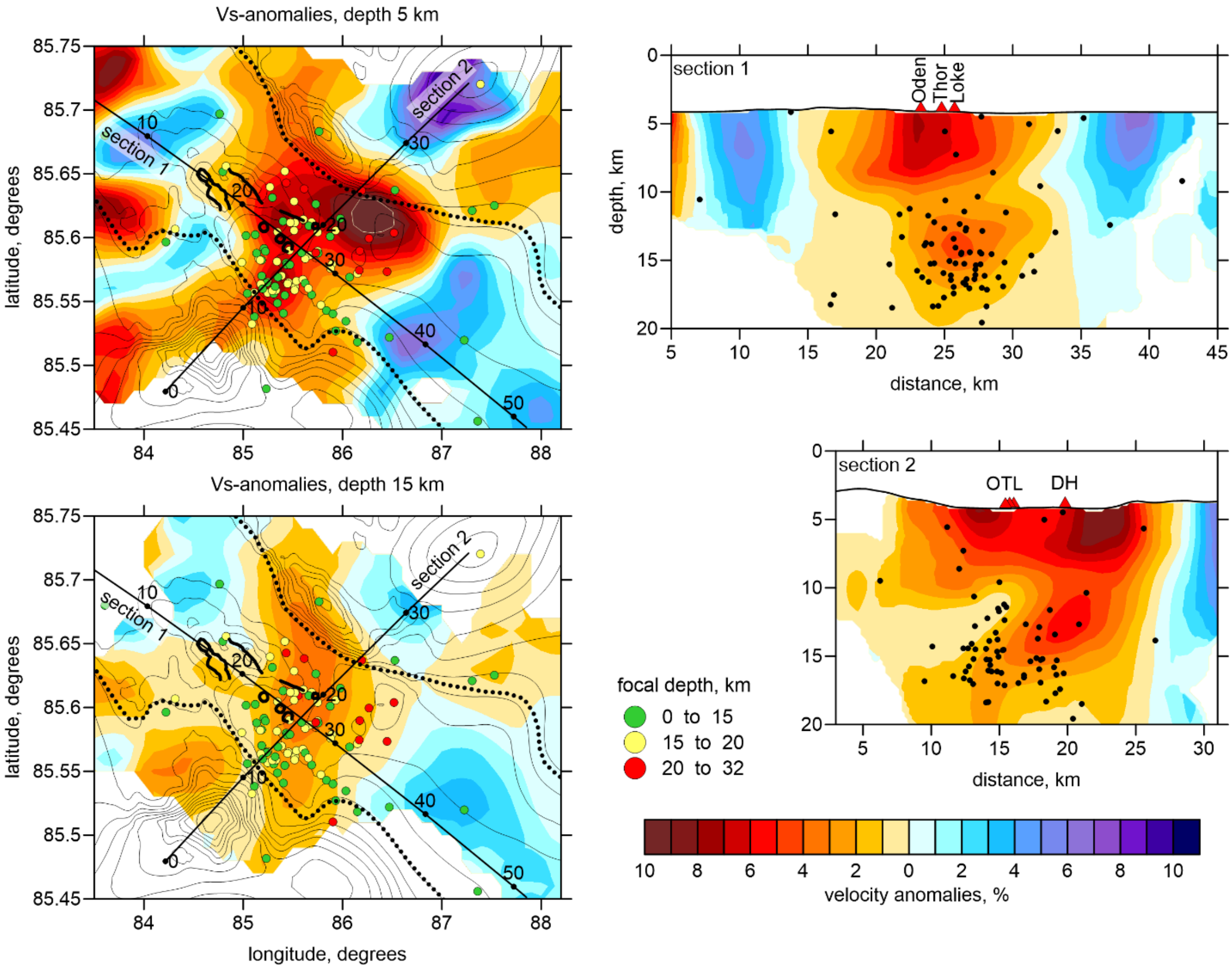


**Figure S4. The resulting distributions of the Vs anomalies** in two horizontal and two vertical sections. Colored dots in the horizontal sections indicate the locations of earthquakes ranged by depth. Dotted lines indicate the borders of the rift valley. The major volcanic structures are highlighted with solid black lines. The bathymetry is presented by thin contour lines at every 100 m. In the vertical sections, black dots indicate the locations of the events at the distances less than 7 km. Red triangles depict the volcanic structures (OTL – Oden, Thor, Loke; DH – Duque's Hills).

In the context of this study, we find it important to present the distributions of the Vp/Vs ratio in the main paper (Fig. 2), as it was found in many studies that this parameter is

sensitive to the content of fluids and melts (23). In our case, the Vp/Vs ratio is derived by division of the resulting absolute velocities of the P and S waves. The adequacy of this method is checked by a series of synthetic tests.

Synthetic modeling is an important stage of the tomography workflow as it gives estimates for the spatial resolution of the recovered structures, as well as optimal values for the inversion controlling parameters. In our case, in synthetic modeling, we simulate the same workflow as used to process the experimental data. The synthetic velocity model is defined as a superposition of the 1D absolute reference velocity and 3D anomalies. The synthetic travel times are calculated for the same stations and source distributions as derived after obtaining the main tomography model. Then we "forget" the coordinates and origin times of the sources and perturb the arrival times with realistically distributed noise with the average deviations 0.05 s and 0.1 s for the P and S waves. The recovery procedure is identical to the case of experimental data inversion including the step of preliminary source locations using the grid-search method. While performing the recovery of the synthetic model, we adjust the main inversion parameters to obtain the best quality of reconstruction; these parameters are then used for the processing of the experimental data.

Here we separately explore the horizontal and vertical resolution by defining the checkerboard anomalies in map view or in vertical sections. In Fig. S5, we present an example of the horizontal checkerboard with the anomalies of 7 km size separated by empty intervals of 3 km width. These dimensions are compatible with the anomaly sizes obtained in the main model. The amplitudes of the anomalies were ±8%, and were opposite for the P and S models to enable strong variations of the Vp/Vs ratio. We see that in most part of the study area both the P and S wave velocity anomalies are correctly recovered. It is important that the Vp/Vs ratio, which is obtained by a simple division of the derived absolute P and S wave velocities, is correctly recovered in this case, which demonstrates the adequacy of this method.

The vertical resolution in the local earthquake tomography is usually poorer than the horizontal resolution because of the fundamental trade-off between the velocity and source parameters; therefore, we assess it using another series of tests shown in Fig. S6. In this case, we defined the synthetic anomalies with the size of 8 km and 2 km spacing along two vertical sections, the same as used for presenting the main results. The change of the anomaly sign occurs at the depth interval of 8-10 km b.s.l., or ~4 km below the seafloor. Across the section,

the anomalies remain unchanged and have a width of 8 km. The noise level, the amplitudes of anomalies and other conditions of the test performing were identical to the horizontal checkerboard test described in the previous paragraph. This test shows that with the existing data we are able to recover the transition zone at ~4 km below the seafloor, which is important for interpretation of the main results.

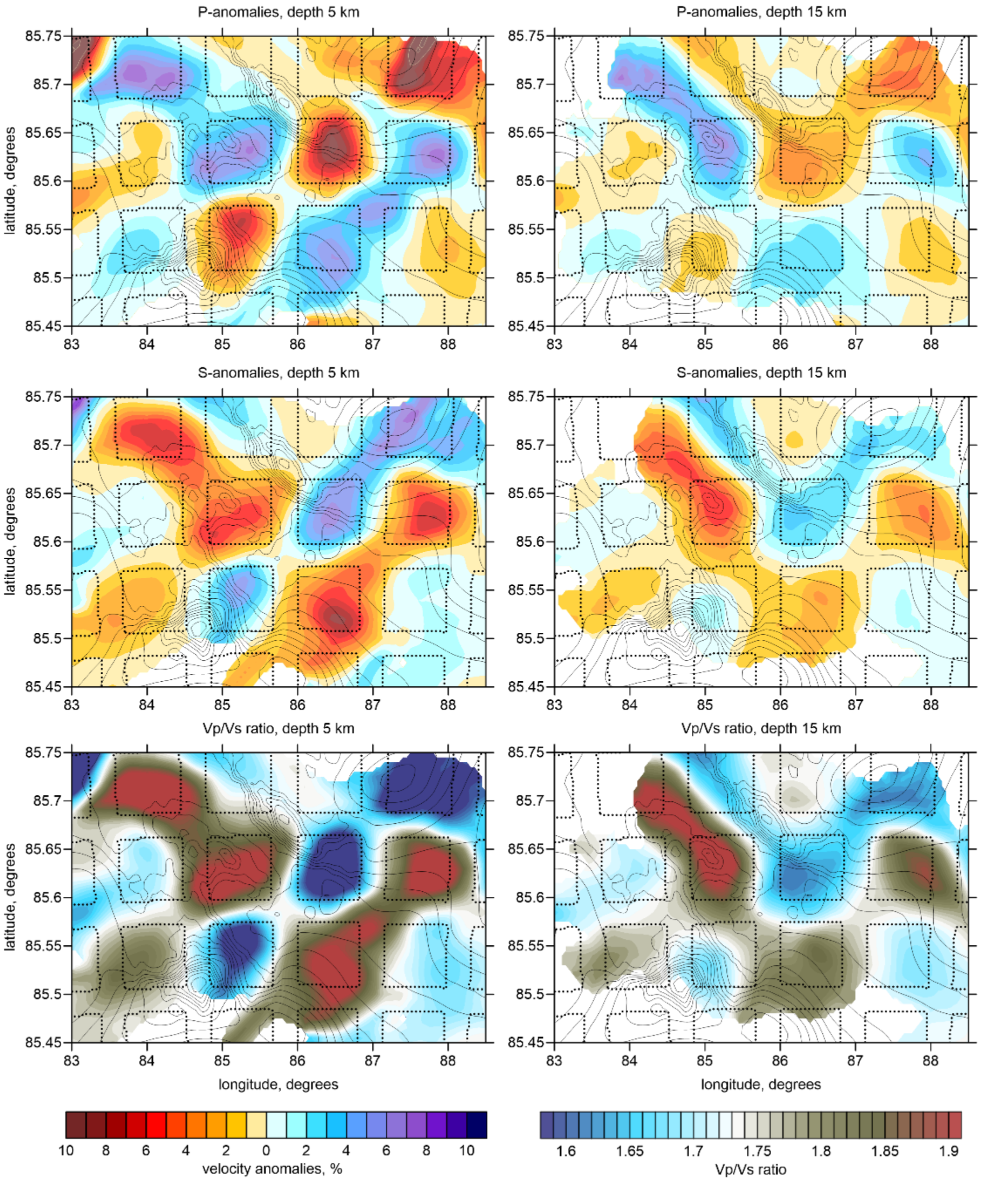


**Figure S5. Checkerboard test with anomalies of 7+3 km size and empty intervals.** The distributions of the recovered anomalies of Vp and Vs, and the Vp/Vs ratio are presented in two horizontal sections. The shapes of the synthetic anomalies are highlighted with the dotted lines. The bathymetry is presented by thin contour lines at every 100 m.

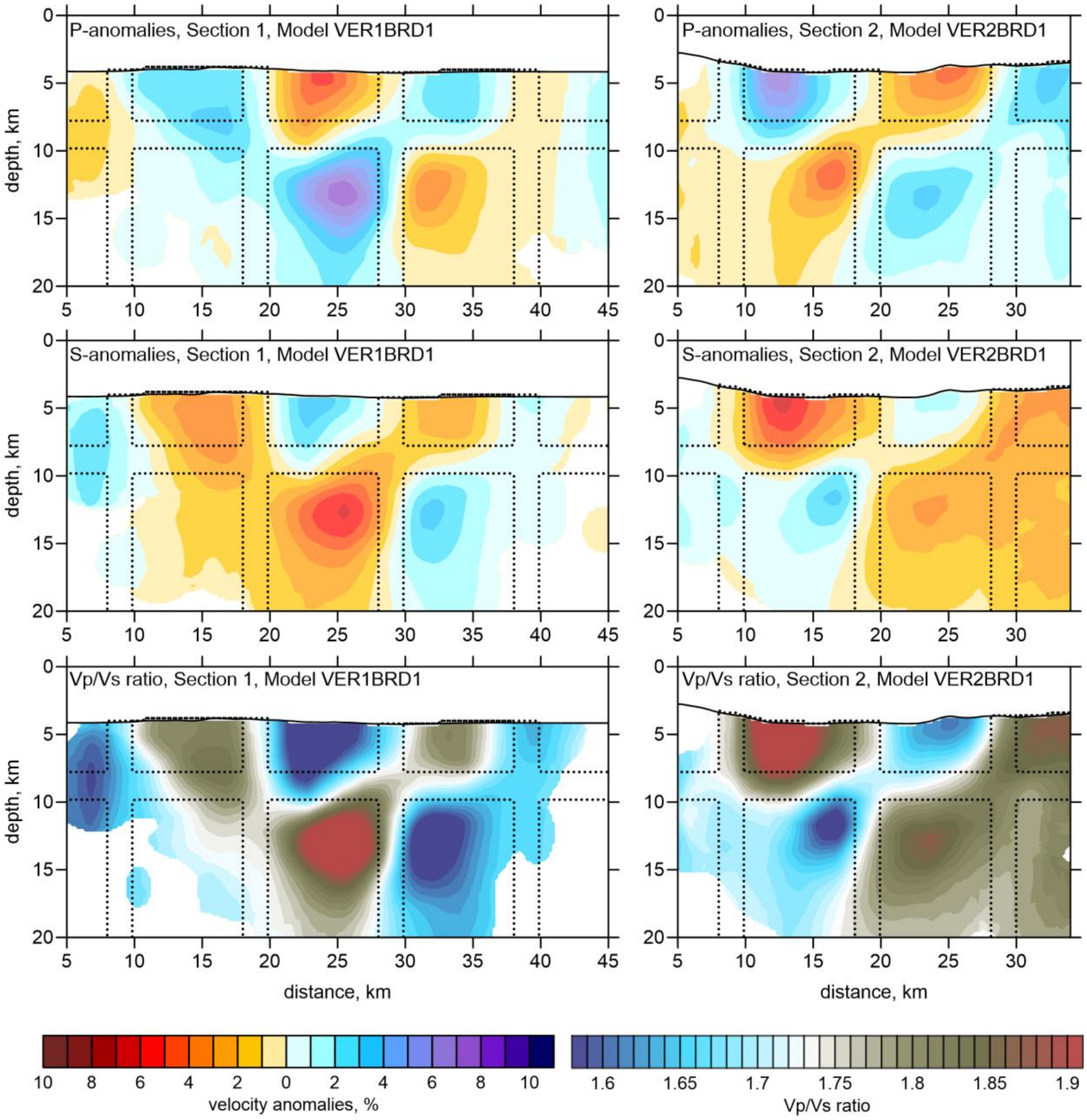


**Figure S6. Synthetic test for checking the vertical resolution.** The anomalies are defined along each of the two vertical sections used for presenting the main results (Figure 2). The shapes of the synthetic anomalies are highlighted with the dotted lines. The distributions of the recovered anomalies of Vp and Vs, and the Vp/Vs ratio are presented in the vertical sections

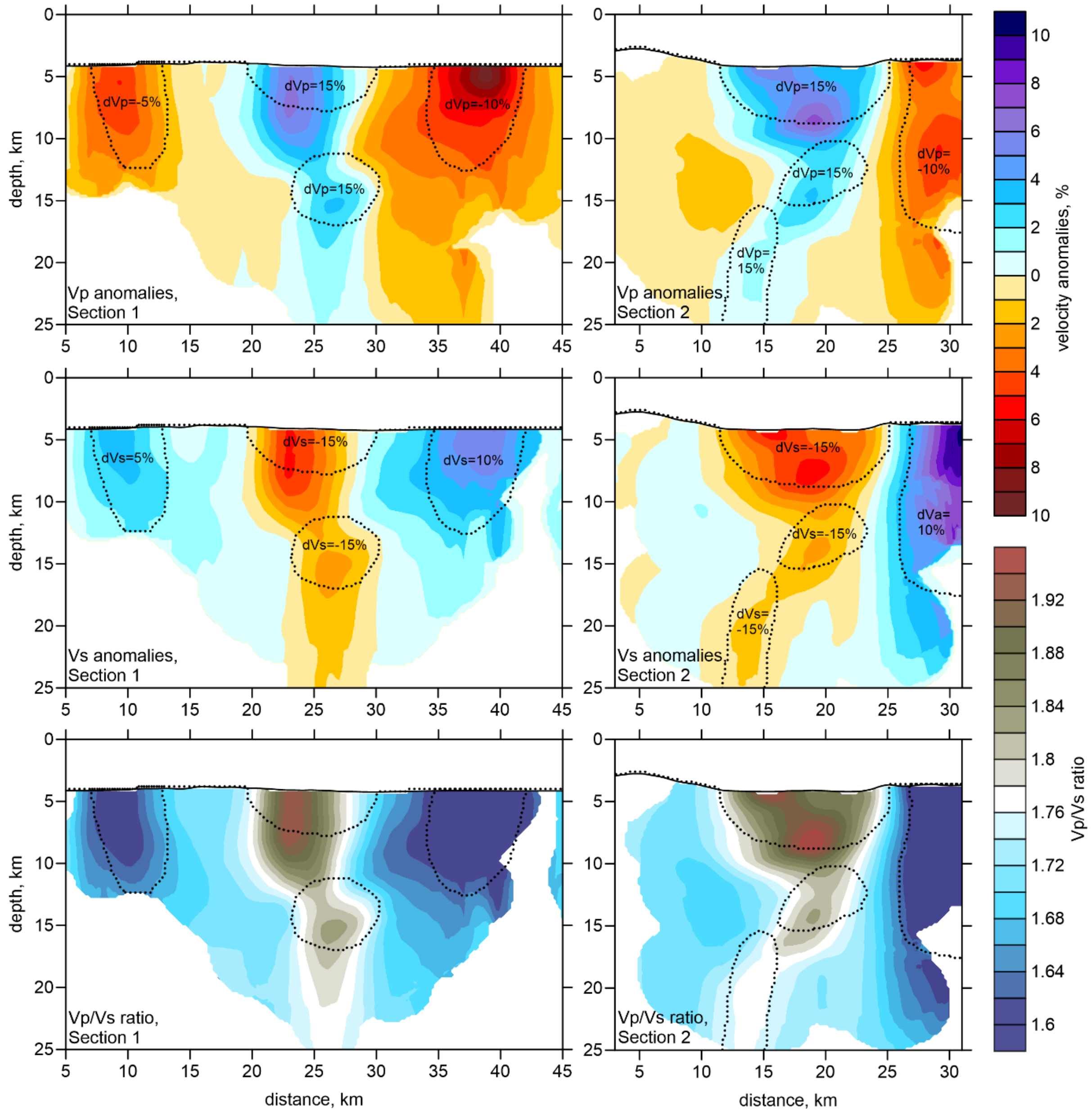


Figure S7. Synthetic tests with free-shaped anomalies defined along sections 1 and 2. The shapes of the initial anomalies are depicted with dotted lines; the amplitudes of the P and S wave anomalies are indicated. The resulting distributions of the dVs, dVs and Vp/Vs ratio are presented in the corresponding vertical sections.

Additional verification was performed using a series of tests with realistic shapes of synthetic anomalies. In these tests, the initial anomalies were defined in a series of closed polygons in a vertical section. In the direction across the section, the thickness of anomalies in all presented tests was equal to 6 km. In Fig. S7, we present two tests with synthetic

models defined along vertical sections 1 and 2. As in the case of experimental data inversion, the values of the P and S wave velocity anomalies have the opposite signs that enabled strong variations of the Vp/Vs ratio in the initial model. The recovery results are shown for the Vp and Vs anomalies, as well as for the Vp/Vs ratio. It can be seen that all the anomalies are recovered at correct locations; however the structures below 10 km depth appear to be smeared. In particular, two anomalies below the rift valley, which have similar magnitudes of the anomalies in the original models, have considerably different amplitudes in the recovered models. The fact of stronger amplitude reducing for the deeper structures during the tomography inversion should be taken into account when interpreting the results.

There might be a concern about the capacity of the tomography to resolve the shallow structures in the rift valley and how these structures affect the source locations. To explore these issues, we considered three models presented in Fig. S8. In these models, we defined different shallow structures: thick upper layer with the amplitudes of ±15% in A, thin upper layer with the amplitude of ±25% in B, and no shallow anomaly in C. In all cases, the "true" coordinates of sources and the starting velocity models were identical. The recovery results show that the cases A and B with thick and thin uppermost layers are hardly distinguishable. In both cases, the reconstructed anomalies propagates down to ~8 km depth. It is important that the differences in the upper structures do not strongly affect the reconstruction of the deeper anomaly. Note also that the differences in the velocity models do not dramatically affect the distributions of the events.

Each of the synthetic tests gives a possibility to assess the accuracy of source locations taking into account the trade-off between the velocity and source parameters and realistic noise in the data. Fig. S9 presents an example of event mislocations the during the synthetic test with realistic anomalies shown in left column in Fig. S7. It can be seen that in the starting 1D model, the mean error of source locations is equal to 4.77 km. After five iterations of tomographic inversions, it is reduced to 3.96 km. Such significant mislocations cause some smearing of the velocity anomalies; nevertheless, the general structures in this model are recovered correctly. The same effect is expected to take part in the results of experimental data inversion.

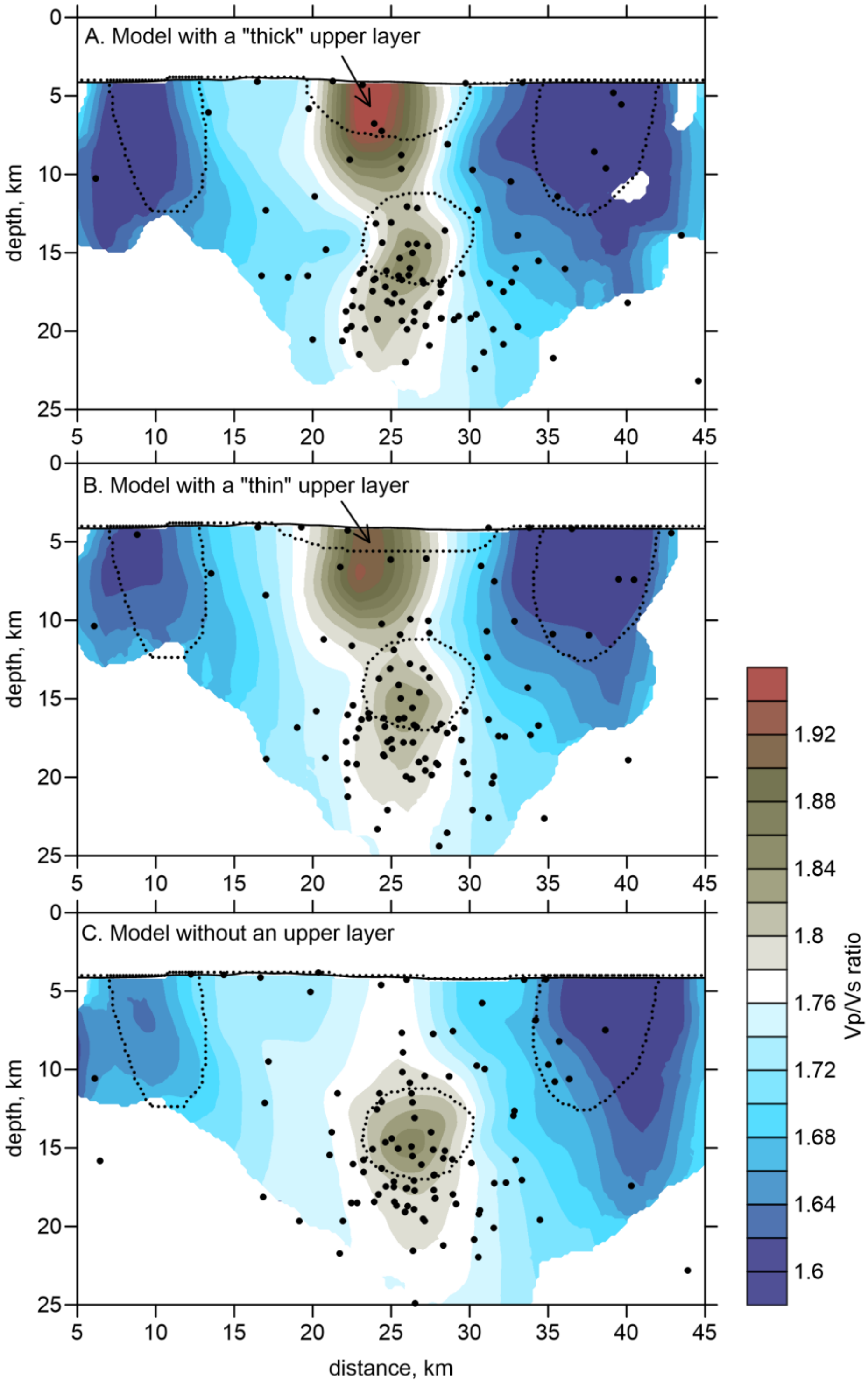


Figure S8. Synthetic tests showing the sensitivity of the model for recovering the shallow anomalies in the rift valley. The initial free-shaped synthetic anomalies depicted by the dotted lines and the recovered Vp/Vs ratio are presented in vertical Section 1. Black dots depict projections of the relocated seismic events.

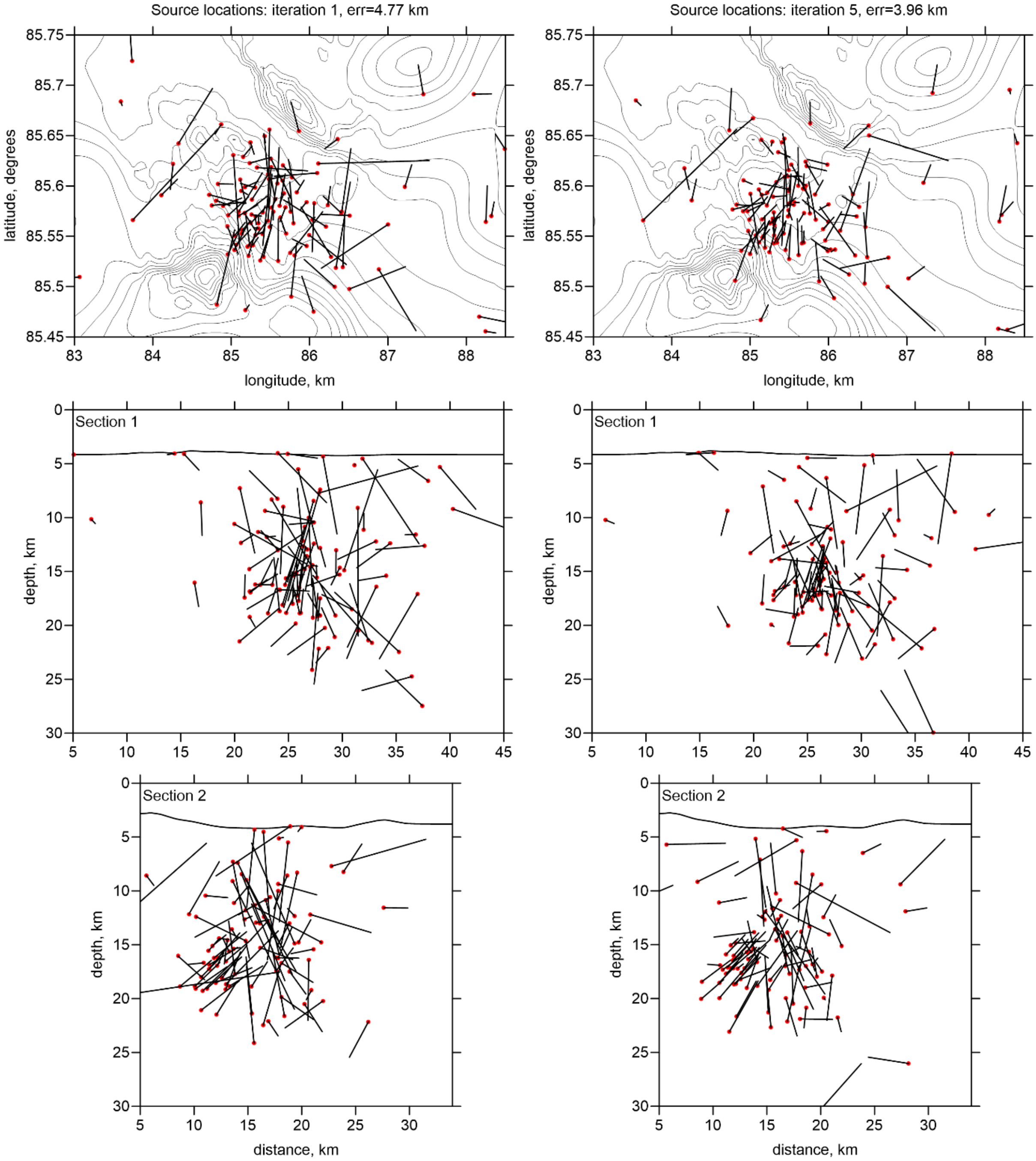


Figure S9. The results of source relocations in synthetic modeling (model in left column in Figure S7) after iterations 1 and 5 shown in map view and two vertical sections. The red dots are the locations of the sources at the corresponding iteration. The ends of the bars indicate the true locations of the events. The values of mean source mislocations (err) are indicated above the upper panels.

## 3. Supplementary figures for magmatic-thermomechanical modelling

The details of the numerical model construction are presented in the Method Section. Here, we present some supplementary figures mentioned there.

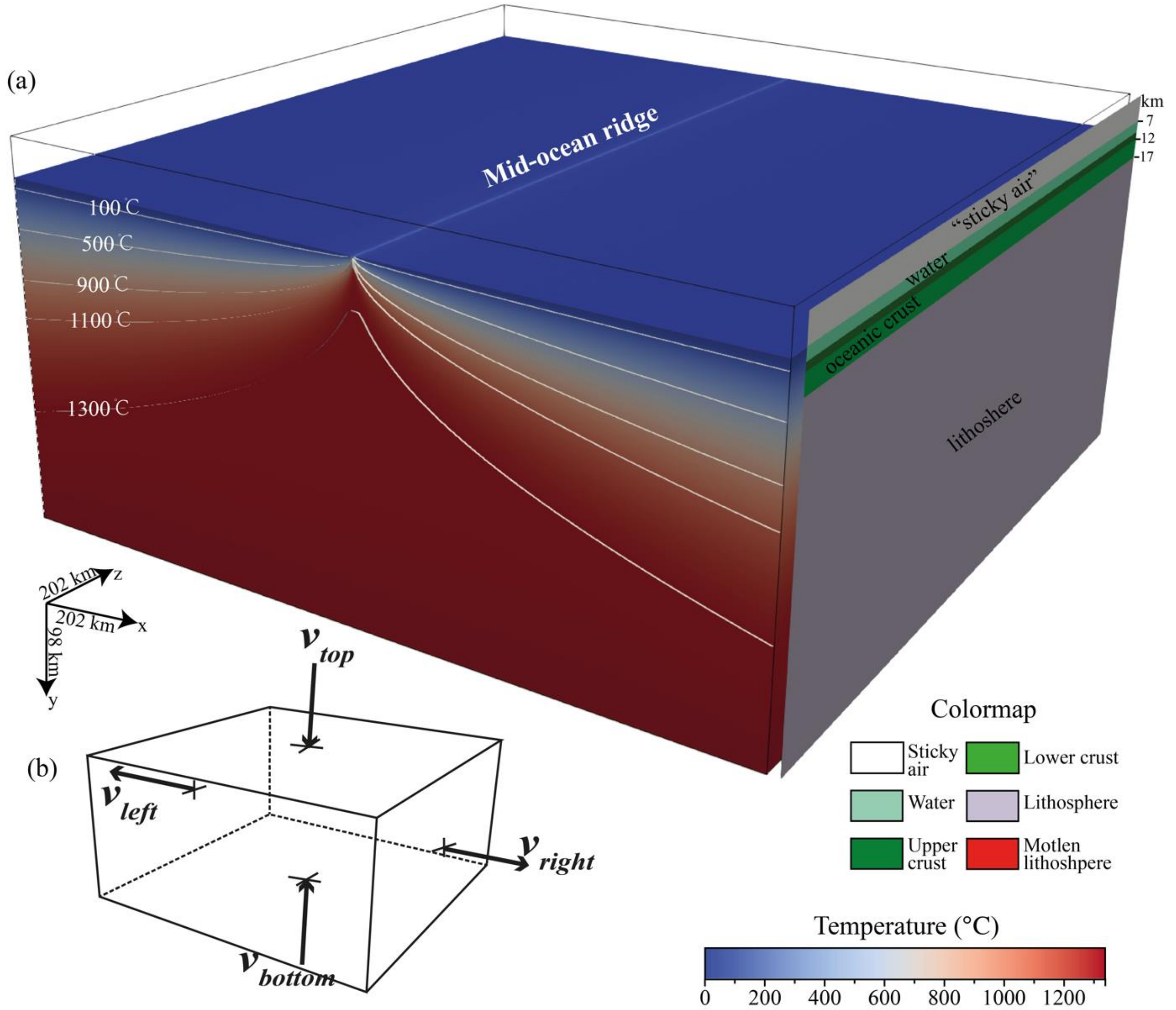


**Figure S10. Initial model setup and boundary conditions for 3D numerical models.** (a) Thermal configuration and composition distribution (right side). (b) Boundary conditions are constant half spreading rate (5 mm/yr) in $x$ direction and compensating vertical influx velocities through the upper and lower boundaries.

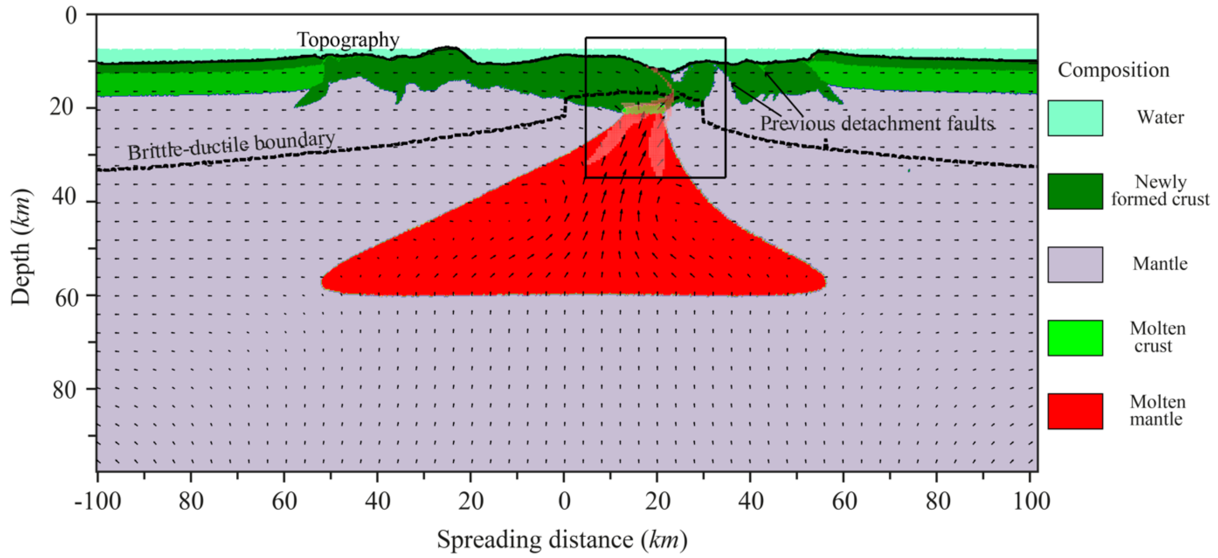


**Figure S11. Composition distribution along the ridge = 63.5 km at evolution time of 10.4 Myr.** Solid black line shows the topography. Dashed black line marks the brittle-ductile boundary. The pink shadow area marks the strain localization, representing the detachment fault. Arrows show the velocity. Black square marks the area studied in the Fig. 3.

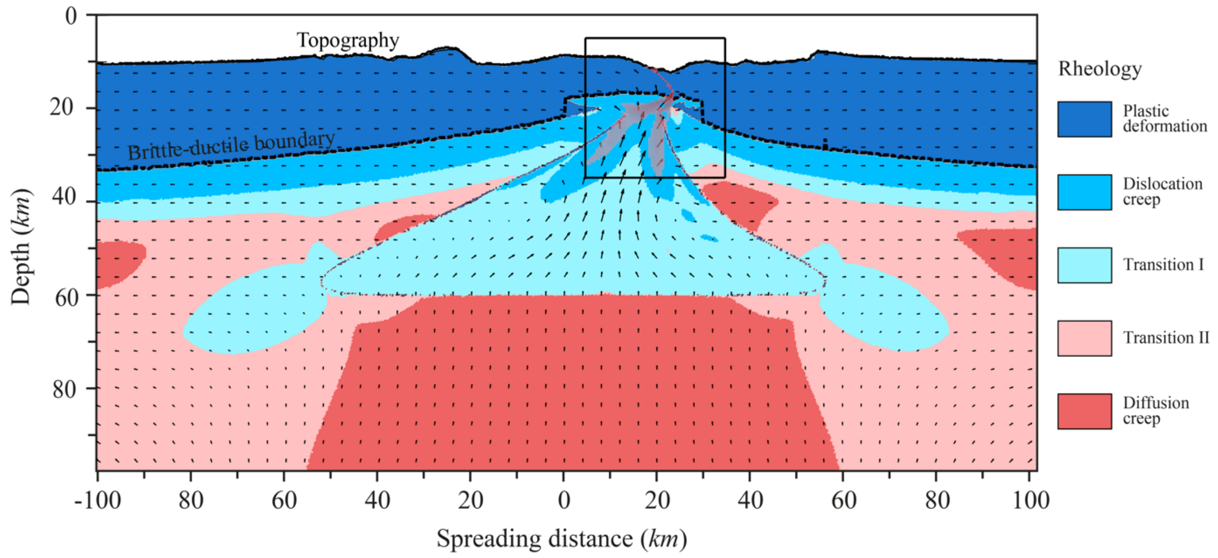


**Figure S12. Rheological mechanism along the ridge = 63.5 km at evolution time of 10.4 Myr.** Molten crust is accumulated beneath the spreading ridge, resulting in the thinned brittle/plastic deformation.

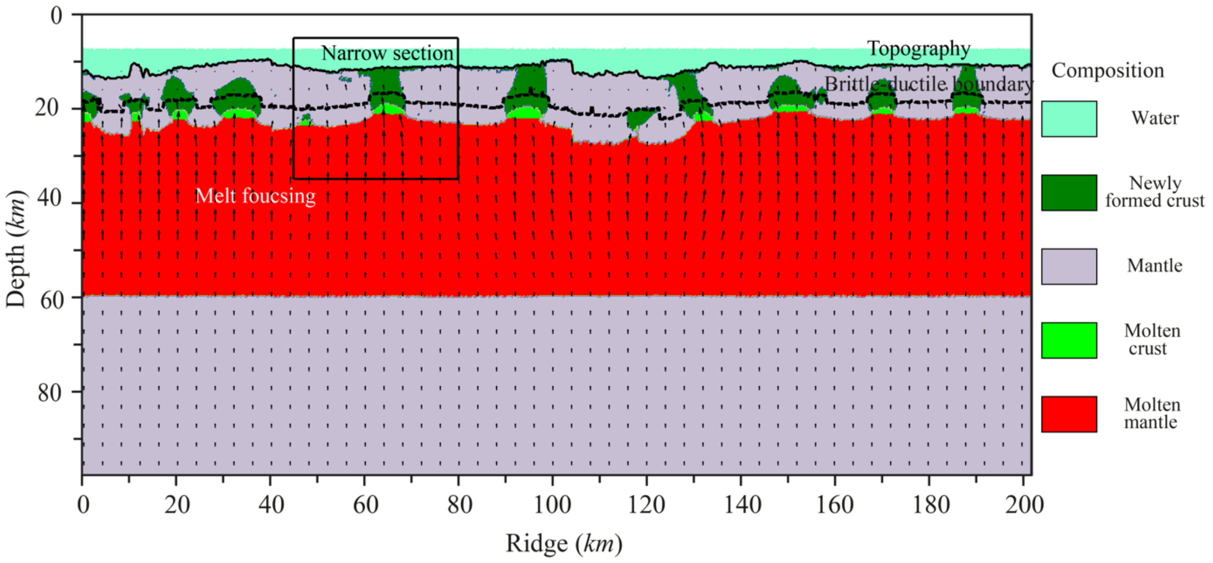


**Figure S13. Composition distribution along spreading ridges at evolution time of 10.4 Myr.** The ultraslow spreading rate and low mantle potential temperature lead to a very low magma supply and narrow magmatic sections.

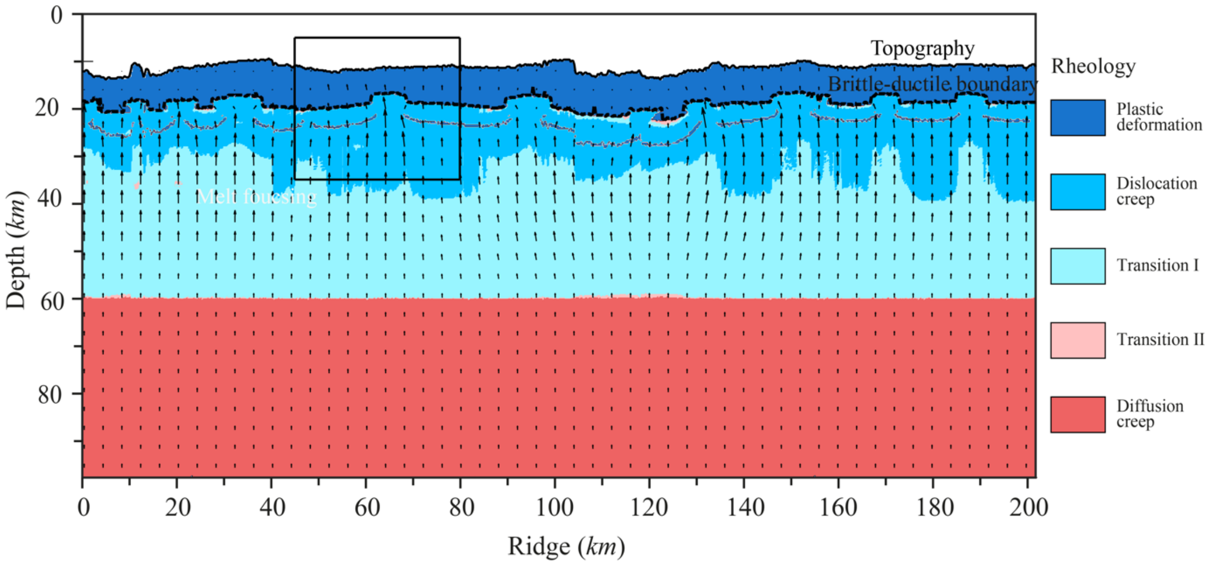


**Figure S14. Rheological mechanism along spreading ridges at evolution time of 10.4 Myr.** The very low magma supply results in strong variations of brittle-ductile boundary depth along spreading ridges.

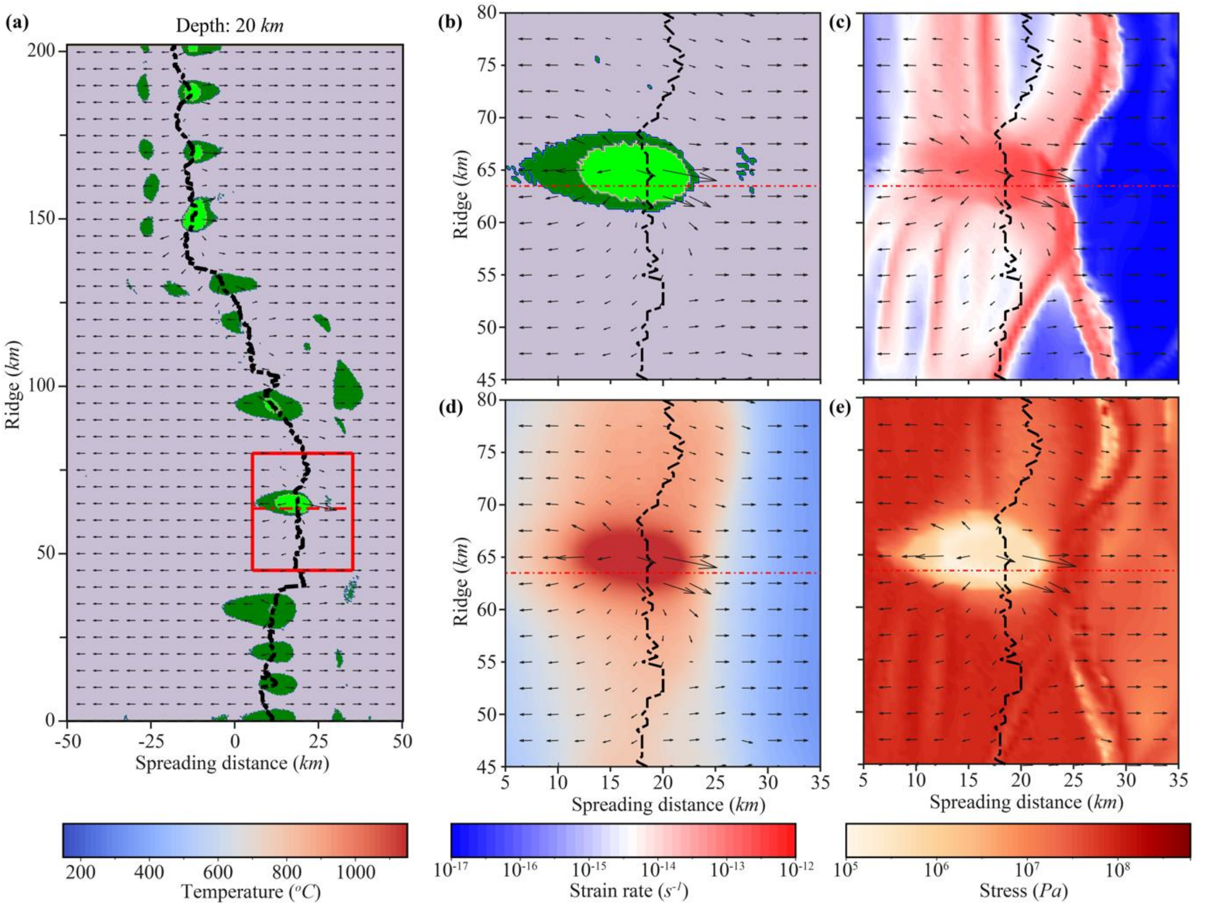


**Figure S15. Horizontal slice at the depth of 20 km at evolution time of 10.4 Myr.** (a) Composition distribution at the depth of 20 km. Red square shows the area plotted in (b-e). Dashed black line marks the spreading ridge. Dashed red line shows the profile location (ridge = 63.5 km) in Fig. 3. (b) Composition. (c) The second invariant of strain rate tensor. (d) Temperature. (e) The second invariant of stress tensor. Arrows show the spreading velocity.

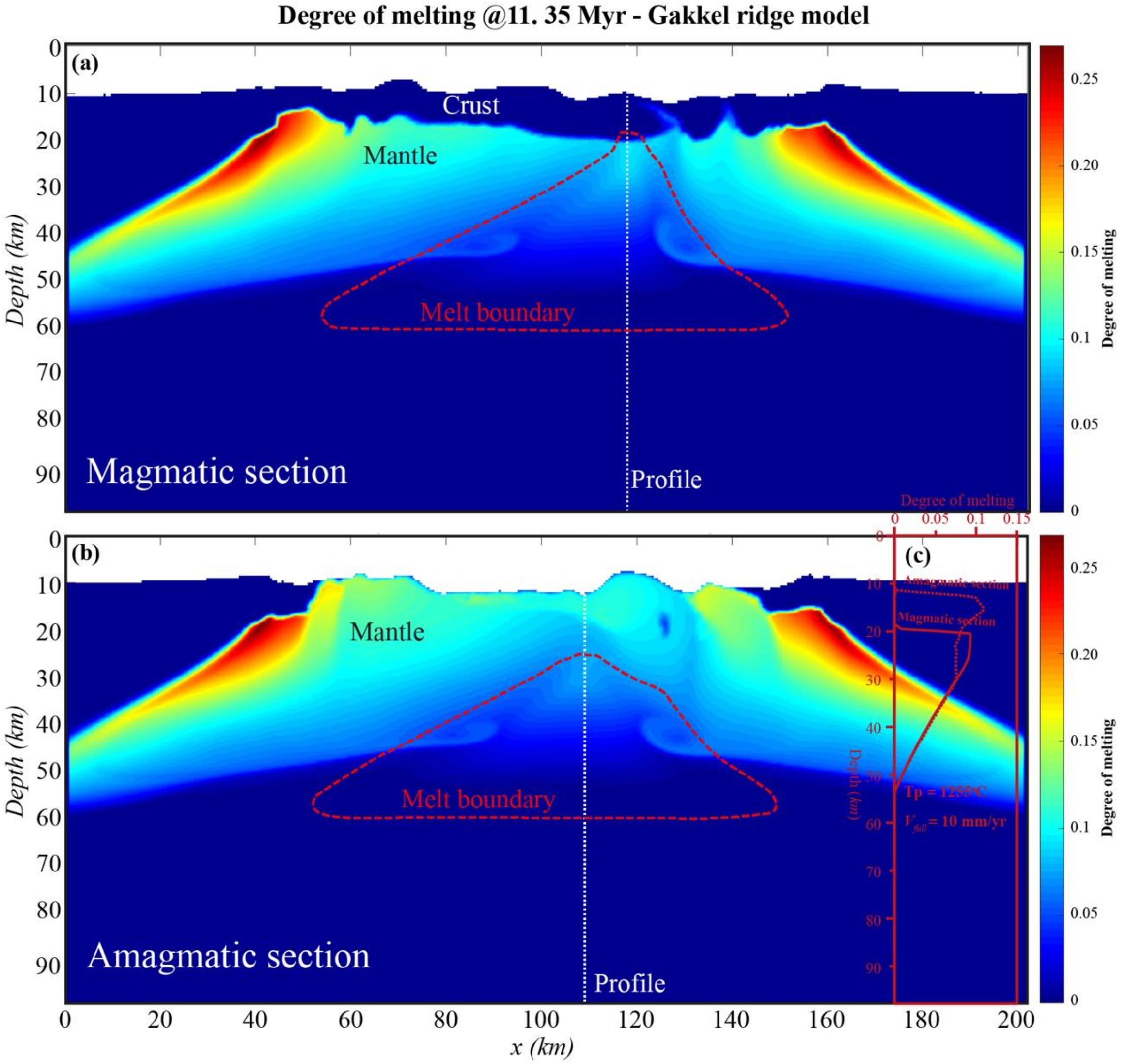


**Figure S16. Distribution of melting degree on mantle markers at both magmatic and amagmatic sections.** (a) The extent of melting at magmatic section. (b) The extent of melting at amagmatic section. (c) The extent of melting along the dashed white lines in (a) and (b). The very high extent of early ridge melting (red to yellow color outside of the melt boundary) in (a) and (b) is caused by the initial thermal configuration.

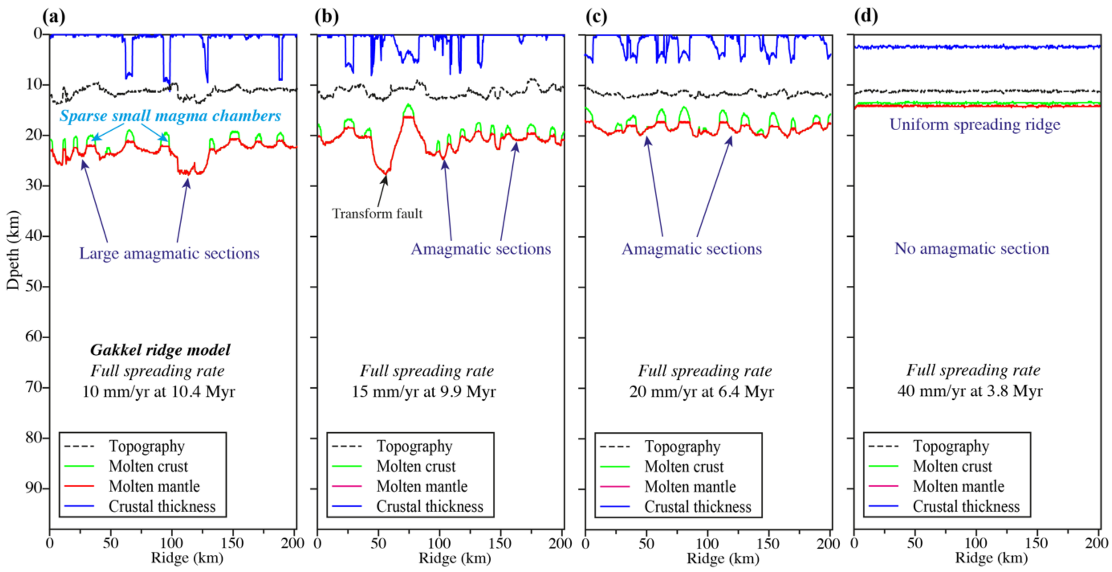


**Figure S17. Numerical models with different spreading rates.** Low mantle potential temperature (1255 ℃) is implemented in these models.

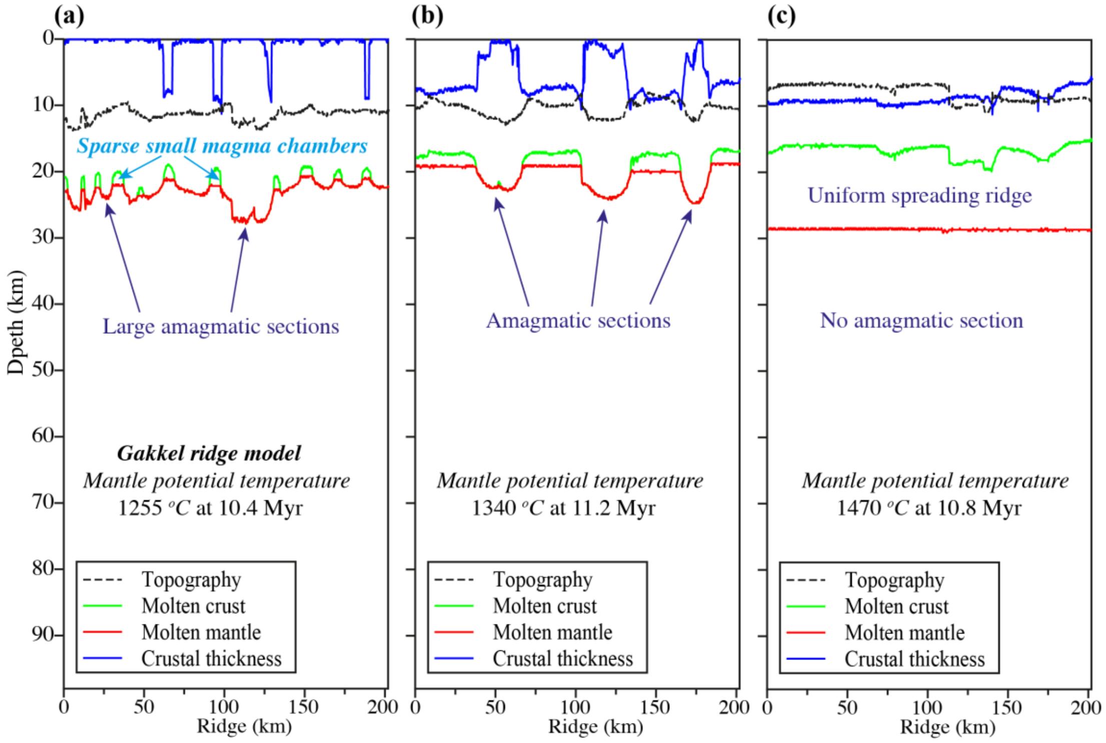


**Figure S18. Numerical models with different mantle potential temperature.** Ultraslow spreading rate (10 mm/yr) is implemented in these models.

**Movie S1: Gakkel_slice.avi.** Composition in 3D is shown with transparent solid mantle. Evolution of vertical slice along z = 63.5 km and horizontal slice along y = 20 km are also shown in the movie.

**Movie S2: Gakkel_surface.avi.** Evolution of crustal thickness, thermal gradient, topography and melt depth in numerical simulation of the ultra-slow spreading.

**Table S2. Physical properties of rocks used in numerical experiments (67-73)**

| Material | Initial density ($\rho_0$: $kg/m^3$) | Thermal conductivity ($W/(m \cdot K)$ $at$ $T$: $K, P$: $MPa$) | $C_0/C_1$ ($MPa$) | $\varphi_0/\varphi_1$ | $\gamma_0/\gamma_1$ | Flow law |
|---|---|---|---|---|---|---|
| Oceanic upper crust | 3000 (solid)<br>2800 (molten) | $1.18 + \frac{474}{T+77}\exp(0.00004P)$ | 10/3<br>3/3 | 0.6/0<br>0/0 | 0/1<br>1/1 | Wet quartzite:<br>$A_D = 4.8 \times 10^{22}, E = 238\ kJ/mol,$<br>$V = 0.0\ m^3/mol,\ \sigma_{cr} = 3 \times 10^4\ Pa, n = 3.2$ |
| Oceanic lower crust | 2800 (solid)<br>2800 (molten) | $1.18 + \frac{474}{T+77}\exp(0.00004P)$ | 10/3<br>3/3 | 0.6/0<br>0/0 | 0/1<br>1/1 | Plagioclase $An_{75}$:<br>$A_D = 4.8 \times 10^{22}, E = 238\ kJ/mol,$<br>$V = 0.0\ m^3/mol,\ \sigma_{cr} = 3 \times 10^4\ Pa, n = 3.2$ |
| Newly formed crust | 2800 (solid)<br>2800 (molten) | $1.18 + \frac{474}{T+77}\exp(0.00004P)$ | 10/3<br>3/3 | 0.6/0<br>0/0 | 0/1<br>1/1 | Plagioclase $An_{75}$:<br>$A_D = 4.8 \times 10^{22}, E = 238\ kJ/mol,$<br>$V = 0.0\ m^3/mol,\ \sigma_{cr} = 3 \times 10^4\ Pa, n = 3.2$ |
| Lithospheric-asthenospheric mantle | 3300 (solid)<br>2900 (molten) | $0.73 + \frac{1293}{T+77}\exp(0.00004P)$ | 10/3<br>3/3 | 0.6/0<br>0/0 | 0/1<br>1/1 | Dry olivine:<br>$A_{ds} = 1.1 \times 10^{-16}, E_{ds} = 530\ kJ/mol,$<br>$V_{ds} = 1.4 \times 10^{-5}\ m^3/mol,\ n = 3.2$<br>$A_{df} = 1.5 \times 10^{-15}, E_{ds} = 375\ kJ/mol,$<br>$V_{ds} = 6 \times 10^{-6}\ m^3/mol,\ m = 3$<br>$r = 3\ mm$ |
| Serpentinized mantle inside faults | 3300 (solid)<br>2900 (molten) | $0.73 + \frac{1293}{T+77}\exp(0.00004P)$ | 1/1<br>1/1 | 0.1/0<br>0/0 | 0/1<br>1/1 | Serpentine:<br>$A_D = 5.01 \times 10^{22}, E = 470\ kJ/mol,$<br>$V = 8 \times 10^{-6}\ m^3/mol,\ \sigma_{cr} = 3 \times 10^4\ Pa,$<br>$n = 4$ |

**Table S3. Results of numerical experiments (spreading distance: ~120 km).**

| Model name | Spreading rate $(mm/yr)$ | Bottom temperature $(K)$ | Mantle potential temperature $Tp$ (℃) | Distance between magmatic segments $(km)$ | Results |
|---|---|---|---|---|---|
| Gakk | 10 | 1567 | 1255 | $43 \pm 15$ | Curved ridge with thick brittle layer, sparsely magmatic sections with large distance, low melt fraction |
| Gakk-15 | 15 | 1567 | 1255 | $17 \pm 6$ | Curved ridge, many small magmatic sections, transform fault, |
| Gakk-20 | 20 | 1567 | 1255 | $15 \pm 3$ | Curved ridge, many small magmatic sections with small distance |
| Gakk-40 | 40 | 1567 | 1255 | - | Straight ridge with thin brittle layer, no amagmatic section with uniform thin oceanic crust and flat topography, high melt fraction |
| Gakk-t100 | 10 | 1667 | 1340 | $25 \pm 4$ | Curved ridge with thick brittle layer, alteration of magmatic zone with hot and high topography and amagmatic zone with cold and low topography |
| Gakk-t200 | 10 | 1767 | 1470 | - | No amagmatic section, thick oceanic crust with thick brittle layer, high melt fraction, strong small mantle convection |

**References:**


60. Bruvoll, V., Breivik, A. J., Mjelde, R., & Pedersen, R. B. Burial of the Mohn-Knipovich seafloor spreading ridge by the Bear Island Fan: Time constraints on tectonic evolution from seismic stratigraphy. *Tectonics*, **28**(4) (2009).
61. Sauter, D. & Cannat, M. The ultraslow spreading Southwest Indian ridge. Diversity of Hydrothermal Systems on Slow Spreading Ocean Ridges, *Geophysical Monograph Series, American Geophysical Union* **88**, 153-173, DOI 10.1029/2008GM000843 (2010).
62. Müller, R.D., Sdrolias, M., Gaina, C. & Roest, W.R. Age, spreading rates, and spreading asymmetry of the world's ocean crust. *Geochemistry, Geophysics, Geosystems* **9**(4), https://doi.org/10.1029/2007GC001743 (2008).
63. Reid, I. & Jackson, H.R. Oceanic spreading rate and crustal thickness. *Marine Geophysical Researches* **5**(2), 165-172 (1981).
64. Bown, J.W. & White, R.S. Variation with spreading rate of oceanic crustal thickness and geochemistry. *Earth and Planetary Science Letters*, **121**(3-4), 435-449 (1994).
65. Schlindwein V., Müller C. & Jokat W., Seismoacoustic evidence for volcanic activity on the ultraslow-spreading Gakkel Ridge, Arctic Ocean, *Geophys. Res. Lett.*, **32**(18), L18306, doi:10.1029/2005GL023767 (2005).
66. Frosch, R. A. Underwater Sound: Deep-Ocean Propagation: Variations of temperature and pressure have great influence on the propagation of sound in the ocean. *Science*, **146**(3646), 889-894. (1964).
67. Clauser, C., & Huenges, E. Thermal conductivity of rocks and minerals. In: Ahrens, T.J. (editor), Rock Physics and Phase Relations. *AGU Reference Shelf 3*. American Geophysical Union, Washington DC, 105–126 (1995).
68. Hirth, G., & Kohlstedt, D. Rheology of the upper mantle and the mantle wedge: a view from the experimentalists. In: Eiler, J. (Ed.), Subduction Factor Monograph, vol.138. American Geophysical Union,Washington, DC, pp. 83–105 (2003).
69. Hilairet, N., B. et al. High-pressure creep of serpentine, interseismic deformation, and initiation of subduction. *Science,* **318**, 1910–1913. (2007).
70. Hofmeister, A. M. Mantle values of thermal conductivity and the geotherm from Phonon lifetimes. *Science*, **283**, 1699–1706 (1999).
71. Karato, S., & Wu, P. Rheology of the upper mantle: a synthesis. *Science* **260**, 771–778 (1993)
72. Ranalli, G. Rheology of the Earth (Chapman and Hall, 1995).
73. Turcotte, D. L. & Schubert, G. Geodynamics (Cambridge Univ. Press, 2002).
